# An integrative review on the acceptance of artificial intelligence among healthcare professionals in hospitals

**DOI:** 10.1038/s41746-023-00852-5

**Published:** 2023-06-10

**Authors:** Sophie Isabelle Lambert, Murielle Madi, Saša Sopka, Andrea Lenes, Hendrik Stange, Claus-Peter Buszello, Astrid Stephan

**Affiliations:** 1 AIXTRA—Competence Center for Training and Patient Safety, Medical Faculty, RWTH Aachen University, Pauwelsstraße 30, 52074 Aachen, Germany; 2 Department of Anesthesiology, Uniklinik RWTH Aachen, Pauwelsstraße 30, 52074 Aachen, Germany; 3 Department of Nursing Science, Uniklinik RWTH Aachen, Pauwelsstraße 30, 52074 Aachen, Germany; 4 Fraunhofer Society for the Advancement of Applied Research. Fraunhofer-Institute for Intelligent Analysis and Information Systems IAIS, Schloss Birlinghoven 1, 53757 Sankt Augustin Bonn, Germany; 5 Fliedner University of Applied Sciences, Geschwister-Aufricht-Straße, 940489 Düsseldorf, Germany

**Keywords:** Medical ethics, Health policy, Public health

## Abstract

Artificial intelligence (AI) in the domain of healthcare is increasing in prominence. Acceptance is an indispensable prerequisite for the widespread implementation of AI. The aim of this integrative review is to explore barriers and facilitators influencing healthcare professionals’ acceptance of AI in the hospital setting. Forty-two articles met the inclusion criteria for this review. Pertinent elements to the study such as the type of AI, factors influencing acceptance, and the participants’ profession were extracted from the included studies, and the studies were appraised for their quality. The data extraction and results were presented according to the Unified Theory of Acceptance and Use of Technology (UTAUT) model. The included studies revealed a variety of facilitating and hindering factors for AI acceptance in the hospital setting. Clinical decision support systems (CDSS) were the AI form included in most studies (*n* = 21). Heterogeneous results with regard to the perceptions of the effects of AI on error occurrence, alert sensitivity and timely resources were reported. In contrast, fear of a loss of (professional) autonomy and difficulties in integrating AI into clinical workflows were unanimously reported to be hindering factors. On the other hand, training for the use of AI facilitated acceptance. Heterogeneous results may be explained by differences in the application and functioning of the different AI systems as well as inter-professional and interdisciplinary disparities. To conclude, in order to facilitate acceptance of AI among healthcare professionals it is advisable to integrate end-users in the early stages of AI development as well as to offer needs-adjusted training for the use of AI in healthcare and providing adequate infrastructure.

## Introduction

Artificial intelligence (AI) is associated with the mechanization of intelligent human behaviour^1^, especially to display intelligent human-like thinking and reasoning^2^. AI is a domain of computer science that is involved in the development of technology that is able to excerpt underlying information from a data set and transform them into operative knowledge. This transformation is based on algorithms that could either be predetermined or adaptive^3^. The term AI was coined in 1956 by John McCarthy but is often connected to the now so-called Turing test. The latter being a hypothetical setup to test, whether or not a machine was able to exhibit intelligent behaviour. Many methods—e.g. knowledge graphs or machine learning techniques have been applied to approximate such behaviour; often without reaching applicability due to computational limits^4^. However, computational limits have seemingly been overcome in many applications^5^. With the increased proliferation of novel AI solutions, issues of reliability, correctness, understanding and trustworthiness have come to the forefront. These issues and the expansion into applications not yet covered by AI solutions mean that the potential of AI technologies has not been fully applied yet, and the continuing growth in the development of AI technologies does not cease to promise new perspectives^2^. Many fields that introduced this new form of intelligence in their domains have witnessed a growth in productivity and efficacy^1^. However, the advantages and disadvantages of AI have to be weighed against one another prior to widespread introduction^1^.

The characterization that defines AI as systems that exhibit behaviours or decisions commonly attributed to human intelligence and cognition is widely accepted^2^. The typically necessary components of such decisions include recognition of a complex situation, the ability to abstract, and the application of factual knowledge^6^. Not all components are always present. Not in every case these systems are “learning”^5^. Decisive for the differentiation to classical systems is that AI systems evaluate complex situations individually and are not based on simple a priori known parameterizations with few input variables^5^.

AI developers are trying to apply their technologies in many fields such as engineering, gaming and education^1^. Lately, the development of AI technologies has expanded to medical practice and its implementation in complex healthcare work environments has begun^1,7–11^. Choudhury et al.^12^ have defined AI in healthcare as ‘an adaptive technology leveraging advanced statistical algorithm(s) to analyse structured and unstructured medical data, often retrospectively, with the final goal of predicting a future outcome, identifying hidden patterns, and extracting actionable information with clinical and situational relevance’ (p. 107)^12^. While AI systems can be applied in the supporting functions (e.g. administrative, legal, financial tasks) around healthcare with similar risks and rewards as in other industries, application to the primary functions of healthcare put a higher demand on suppliers due to regulation and possible impact. While otherwise, typical statistical fluctuations might not be acceptable in the healthcare setting, approaches using knowledge graphs or rule-based techniques, even in combination with machine learning, can lead to intelligent systems, that are robust enough to withstand the scrutiny of governing bodies and medical guidelines. Furthermore, refraining from systems that act fully on their own, but offer support to a licensed professional overseeing the actual application, can be made to satisfy legal and regulatory hurdles^1^.

Until now, AI has been established in the healthcare sector with the purpose of proposing efficient and practice-oriented solutions for patients and healthcare providers. In this field, AI is being developed to benefit healthcare professionals such as physicians and nurses in decision-making, diagnosis, prognosis, treatment and relief from physically demanding tasks^1,11,13^. However, they are not being extended to larger settings. Ethical issues, lack of standardization, and unclear legal liability are among the challenges that face the widespread of AI in healthcare today^14^.

Newly introduced change and its implementation are being faced with mixed attitudes and feelings by healthcare professionals^1,13^. Accepting change is not a simple process. Humans are known to resist change in exchange for the comfortable status quo. However, in order to improve efficiency and workflow in the long run, acceptance is a key element to adopting and implementing newly introduced changes such as AI in daily practice^5,15^.

In the context of technology, acceptance is defined as the willingness, intention and internal motivation to use a technology as a result of positive attitudes towards the technology or system^16^. Acceptance of AI systems plays a similar role as with the introduction of all other new tools. However, the less predictable handling of complex situations and the desired human-like behaviour quickly lead to more resistance^15^. On the side of the developers of these systems, acceptability rather than acceptance is studied. This is usually associated with terms like comprehensibility or transparency, which are supposed to lead directly to acceptability^17^. This is applied at the technical and legal level and in decisions about deployment at the management level. The level of acceptance by the user eludes such approaches and should instead be evaluated directly. Only through this step acceptance may be traced back to acceptability^17^.

This integrative review aims to unravel the variety of reported causes for the limited acceptance as well as facilitating factors for the acceptance of AI usage in the hospital setting to date. The assessment and analysis of reasons for distrust and limited usage are of utmost importance to face the increasing demands and challenges of the healthcare system as well as for the development of adequate, needs-driven AI systems while acknowledging their associated limitations. This includes the identification of factors influencing the acceptance of AI as well as a discussion of the mechanisms associated with the acceptance of AI in light of current literature. This review’s findings aim to serve as a basis for further practical recommendations to improve healthcare workers’ acceptance of AI in the hospital setting and thereby harness the full potential of AI.

## Results

**Fig. 1:**
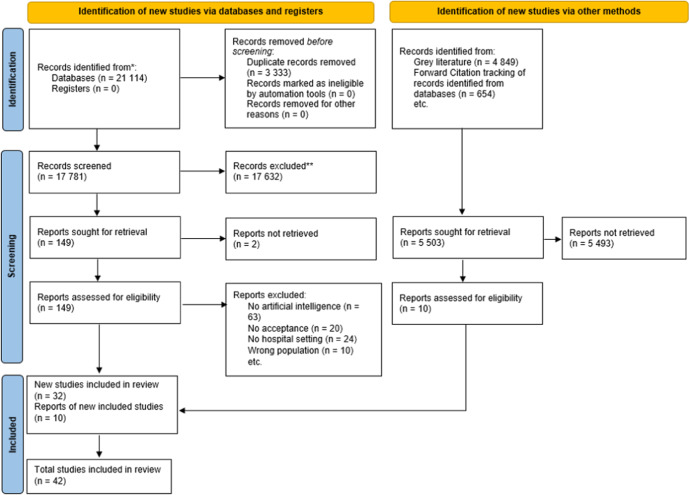
PRISMA 2020 flow diagram.

As shown in Fig. 1, the database search generated (*n* = 21,114) references. After deleting duplicates, sorting the articles according to the inclusion and exclusion criteria and applying forward citation tracking, a total of (*n* = 42) articles were included in this review.

Most studies were carried out in Europe (*n* = 13) followed by Asia (*n* = 12) and North America (*n* = 10). Further studies were conducted in Africa (*n* = 4) and Australia (*n* = 2). One international study was carried out in 25 different countries. There were qualitative studies (*n* = 18), quantitative studies (*n* = 16) and studies with a mixed-method approach (*n* = 8). All study participants were healthcare professionals working in a hospital setting. Instruments for data collection included interviews and surveys. The sample size of included studies varied between 12 and 562 and the age of participants ranged between 18 and 71 years (age reported in 21 studies). The average score for critical appraisal measured by means of the MMAT was 4.45 (Table 3).

In the following paragraphs, the results of our findings will be presented with reference to the UTAUT model. Table 1 represents a summary of the results in relation to the four main UTAUT aspects.

**Table 1:** Summary of the results in relation to the four main UTAUT aspects.

The four main UTAUT aspects	Results pertaining to each of the aspects
Performance expectancy	- alerts and medical errors - time and workload - accuracy of AI technologies
Effort expectancy	- transparency and adaptability of the system - the system’s characteristics - training to use the system
Social influence	- influencing effects on decision making - communication in the workplace
Facilitating conditions	- legal liability - organizational culture - organizational infrastructure

### Performance expectancy

Heterogeneous findings are reported with respect to healthcare professionals’ confidence that using AI systems will benefit their performance. In the included studies, results reflecting on performance expectancy were reported with regards to alerts and medical errors, and the accuracy of AI technologies.

In three studies handling the adoption of clinical decision support systems (CDSS), participants indicated that in acute hospital settings, CDSS reduced the rate of medical errors through warnings and recommendations^18–20^. On the other hand, in one study about the barriers to adopting CDSS, participants reported that CDSS induced errors in emergency care settings^21^. AI in neurosurgery was the topic of a study in which 85% of 100 surgeons, anaesthetists and nurses considered alerts to be useful in the early detection of complications^22^. Similar results were reported in a study that evidenced that 90% of participating pharmacists and physicians (36/40) considered that an automated electronic alert improved the care of patients with acute kidney injury^23^. These findings were also supported in further studies about healthcare professionals’ perception of CDSSs in which participants described alerts as effective in drawing attention to key aspects^18,24^. Nevertheless, in one study about barriers to the uptake CDSSs, respondents found the number of alerts to be excessive^25^. In addition, in three studies, participating physicians and nurses mentioned fatigue resulting from frequent alerts^26–28^. Moreover, Kanagasundaram et al.^29^ reported that some physicians dismissed alerts^29^.

Healthcare professionals’ estimation of the accuracy of technologies based on AI was inconsistent. Results of a study showed that 22.5% of staff from a radiology department (*N* = 118) deemed AI-based diagnostic tools to be superior to radiologists in the near future^30^. However, only 12.2% (*N* = 204) claimed that they would “always use AI when making medical decisions in the near future”^30^. A study by O’Leary in which doctors, nurses and physiotherapists’ appraisal of the diagnostic abilities of AI support systems in view of rare or unusual diseases was investigated, found that 82% of respondents (*N* = 19) considered the tool to be useful^31^. Jauk et al.^32^ concluded that 14.9% of participating doctors and nurses (7/47) did not believe that a machine learning system could detect early-stage delirium^32^. Similarly, 49.3% of physicians (277/562) in a study assessing the use of AI in ophthalmology indicated that the quality of the system was difficult to guarantee^33^. In three studies that assessed healthcare professionals’ attitudes towards CDSS, findings implied that participants doubted the CDSS and diagnostics systems’ accuracy as they considered the quality of resulting information to be insufficient for decision making^21,28,34^. In another study on the same topic, physicians reported that CDSSs are useful but that their functions are limited^27^. Similarly, technical issues that might affect an AI system and render its results inconsistent were found to negatively affect the performance expectancy of physicians, nurses and operating room personnel and resulted in frustration^10,19,22,25,34^. In addition, in a study that investigated their attitudes towards potential robot’s use in a paediatric unit, nurses reported that they were sceptical of the abilities of the system^35^. Similarly, nurses stated in a study about adopting a CDSS that technical issues might affect the system and render its results inconsistent^26^.

Nevertheless, in qualitative studies on the topics of implementing AI in radiology and integrating a machine learning system into clinical workflow, physicians and nurses perceived AI to be accurate and based on sufficient scientific evidence in terms of diagnostics, objectivity and quality of information^8,32,35,36^.

### Effort expectancy

Heterogeneous findings were also reported with respect to how easy the users believe it is to use a system. In the included studies, results reflecting on effort expectancy were reported in regard to time and workload, transparency and adaptability of the system, the system’s characteristics and training to use the system.

Efficiency with respect to time and workload was a recurrent theme in several included articles^10,18,20,26,35–38^. In a study by McBride et al.^39^ on robots in surgery, physicians were concerned about an increased operative time in robotic-assisted surgeries, whereas nursing and support theatre staff did not share these concerns^39^. However, in a study about the acceptance of a machine learning predictive system, 89.4% of nurses and doctors (42/47) did not report an increase in workload when using the algorithm in their clinical routine^32^. In a qualitative study about physicians’ adoption of CDSSs, participants reported CDSSs to be time-consuming^37^. Moreover, in a study about the attitude of radiologists towards AI, 51.9% of respondents (*N* = 204) appraised AI-based diagnostic tools to save time for radiologists^30^. Besides timely invests, McBride et al.^39^ reported that 52.6% of nursing staff (40/76) and 59.6% of medical staff (28/47) showed concerns that robotic-assisted surgery would increase financial pressure^39^.

In a study about the adoption of AI, physicians stated that a lack of transparency and adaptability of a CDSS system or machine learning system aiding diagnostics would negatively affect its adoption^39^. Moreover, participants of a study about the acceptance of a predictive machine learning system, argued that protocols founding the systems should be comprehensive and evidence-based^32^. A tendency to reject the systems was evidenced when participants reported unfamiliarity. This was stated in a study about the experience with a CDSS implemented in paediatrics^29^.

The system’s characteristics also seem to affect the expected effort to use a system which in turn influences its acceptance^40^. Participants of a study about the perception of a CDSS reported that when the system was perceived as intuitive, easily understood, and simple it was highly regarded by participants^41^. However, when the system was complex and required added tasks, such as reported in a study about integrating machine learning in the workflow, it was deemed undesirable^36^. In one study addressing the overall perception of AI by healthcare professionals, at least 70% of respondents (67/96) agreed on each item referring to the ease of use of AI-based systems^42^. However, Jauk et al.^32^ reported that 38.3% of users (18/47) of a machine learning algorithm reported that they were not able to integrate the system into their clinical routine^32^. In a study by Tscholl et al.^43^, 82% of anaesthesiologists (31/36) agreed or strongly agreed with the statement that the technology was “intuitive and easy to learn”^43^. When participants believed the AI-based system was aligned with their tasks, had consistent reporting of values and required minimal time and effort, they welcomed it^43^.

Other studies about CDSS systems reported that participants considered the systems to be inadequate, limited and inoperative in clinical practice^19,38,44^. A standardized CDSS system with clear guidelines seemed appealing to participants who approved of structured systems and commented positively on their ease of use^27,34^. Conversely, in a study about AI in radiology, participants reported that the system lacked standardization and automation and was therefore deemed unreliable^10^.

The importance of training for the successful implementation of AI systems was stressed upon in several studies. In one study referring to a continuative, predictive monitoring system^41^ and two addressing machine learning systems^10,45^, participants reported a lack of experience with the systems which resulted in feeling overwhelmed^38,43,46^. Alumran et al.^47^ observed that about half (53.49%) of nurses (*N* = 71) who did not use an AI system also did not participate in prior training^47^. Half of those receiving one training used the system whereas taking two training courses resulted in the use of the system in 83% of trained nurses. When taking three training courses this percentage increased to 100%^47^.

### Social influence

The description of how much of an effect the opinion of others has on the study participants believing that they should use the AI systems was reported on in several studies. Results of studies reflecting on social influence were reported in regard to the influencing effects on decision-making and communication in the workplace.

In two studies about the acceptance and adoption of CDSS, physicians reported that their decision to use the system was independent of the opinion of supervisors and colleagues^18,24^. However, they reported that patients’ satisfaction with an AI system positively influenced their acceptance^18,24^.

One facilitating factor to the adoption of CDSS systems was believed to be communication between (potential) users of the systems^25^. Some studies pointed out the positive effects of CDSS systems and computerized diagnostic systems on the improvement of interdisciplinary practice and communication^25,36^. Nevertheless, in one study, physicians suggested that CDSS systems may reduce time spent with patients^37^. In view of the use of robotics in pediatrics, nurses emphasized that working with robots would have a negative effect on patients due to a reduction in human touch and connection^35^.

### Facilitating conditions

Healthcare professionals’ views on organizational support to use the system were discussed in several included studies. The main discussion themes on this topic were legal liability, the organizational culture of accepting or rejecting AI systems and organizational infrastructures.

Concerns about legal liability and accountability were raised in several studies. Medical practitioners in a study about a diagnostic CDSS did not have a clear understanding of who would be accountable in case of a system error, which resulted in confusion and fear of the system^48^. Only 5.3% of respondents (*N* = 204) in a study about the attitude of radiologists towards AI stated that they would assume legal responsibility for imaging results provided by AI^30^. In two of the reviewed publications, participants addressed the topic of data protection. They mentioned the importance of maintaining data privacy as a positive aspect in the acceptance of AI systems, especially in CDSSs^24,25^.

In a study about the implementation of AI in radiology, the effect of organizational culture on the acceptance of the system versus the resistance to change was discussed. Several participants mentioned structuring the adoption on the system by selecting champions and expert groups^10,32^. However, in another study reporting on a wound-related CDSS, some nurses preferred to base their behaviour on their own decision-making process and feared that their organization was forcing them to do otherwise^34^.

The importance of an adequate infrastructure to implement AI systems as well as space and monetary resources were stressed^18,49^. The fact that AI systems oftentimes are in need of high-speed internet with a stable connection rendered them inoperable in the face of unavailability of good internet conditions which was expressed as problematic by some participants^41,42^. Additionally, in a study by Catho et al.^37^ on the adoption of CDSSs, several participating physicians highlighted the importance of providing technical support to users in order to increase acceptance of the system^37^.

### Gender

Only three studies investigated whether there was an effect of gender on acceptance. None of them found significant effects^50–52^.

### Age

With respect to age, three studies investigated whether there was an effect of age on the use of AI. Two studies did not observe an effect^50,52^. Walter et al.^53^ found that 55.8% of younger participants claimed that they would use automated pain recognition. In the older age group, only 40.4% of respondents reported that they would use the system (*N* = 102)^53^.

### Experience

Stifter et al.^51^ reported that participants with less than one year of experience reported higher levels of perceived ease of use, perceived usefulness and acceptability of a CDSS than those with more than one year of experience, although the last was statistically non-significant^51^. In contrast, So et al.^42^ reported a statistically significant positive correlation between working experience and use of AI^42^. Similarly, Alumran et al.^47^ observed that an increase in working experience correlated with the use of an electronic triage system^47^.

### Voluntariness of use

Participants of the included studies talked about the fear of AI replacing healthcare professionals as well as a loss of autonomy related to the use of AI. These two aspects could have an effect on the voluntariness to use AI systems.

Participants raised the concern that AI may replace healthcare professionals in their duties at some point. Among respondents, 54.9% reported that physician candidates should opt for “specialty areas where AI cannot dominate”^39^. Similarly, 6.3% of respondents expected AI to completely replace radiologists in the future^39^. In a study by Zheng et al.^33^, 24% of respondents (135/562) denied the claim that AI would completely replace physicians in ophthalmology^54^. Nevertheless, 77% of physicians and 57.9% of other professional technicians believed that AI would at least partially replace physicians in ophthalmology^54^. These findings were also replicated in two qualitative studies that explored the acceptance and adoption of CDSS in which physicians vocalized their fear of being replaced by the systems, and of their work becoming outdated^18,47^.

In a study about confidence in AI, physicians revealed a fear of loss of autonomy in stressful situations^2,47^. Nurses who participated in a study about the potential use of robots in paediatrics’ units expressed the concern that robots may limit the development of clinical skills^29^.

In a study assessing the acceptance of a CDSS in neurosurgery, senior physicians and nurses suggested that junior colleagues should refer to them for guidance and final decisions and not to an AI-based system^28^. They feared that blindly following the recommendations of AI-based systems may negatively impact decision-making processes^28^. Similarly, in a study about CDSS in electronic prescribing, junior nurses claimed that they preferred to seek advice from senior nurses instead of an AI-based system, especially in situations in which the system was deemed complex^35^. In addition, in two studies about the acceptance of two different CDSS systems, junior physicians were more open to the use of AI systems than their seniors^25,48^.

## Discussion

The present review included 42 studies and sought to integrate findings about the influencing factors on the acceptance of AI by healthcare professionals in the hospital setting. All findings and evidence were structured with reference to the UTAUT model^40^. Based on the included studies (*N* = 42), acceptance was primarily studied for CDSSs (*N* = 21).

An important factor that could affect the acceptance of AI in healthcare is safety. Different AI systems could lead to different risks of error occurrence which affect the acceptance of the system among healthcare professionals. Although it can be stated that AI-based prediction systems have shown to result in lower error rates than traditional systems^55,56^, it may be argued that systems taking over simple tasks are deemed more reliable and trustworthy and are therefore more widely accepted than AI-based systems operating on complex tasks such as surgical robots. Furthermore, Choudhury et al.^3^, who studied the acceptability of an AI-based blood utilization calculator argued that AI-based systems are often based on data from a norm-typical patient population; however, if the system is applied to unanticipated patient populations (e.g. patients with sickle cell disease), the AI-based recommendation may become inadequate. Such a sample selection bias may not only endanger patient safety but is also likely to increase levels of scepticism about performance expectancy resulting in decreased acceptance among healthcare professionals^3,57^. Moreover, the safety of a system might be affected by technical complications that may influence the quality of the system’s output and therefore limit healthcare professionals’ trust in the system^58,59^. Besides technical complications, insufficient data and information may compromise the accuracy and validity of AI output^60^. By consequence, ensuring high-quality input data as well as ensuring that the system is applied to the anticipated patient population is of utmost importance to AI-based systems’ acceptance^60^.

Additionally, another aspect of safety that was reported to affect effort expectancy and therefore acceptance, is the degree of alert sensitivity of an AI system^61^. The phenomenon of alarm fatigue which refers to “characteristics that increase a clinician’s response time and/or decrease the response rate to a clinical alarm as a result of too many alarms”^62^ is a result of the AI system and could affect the safety of patient care. To sum up, overly sensitive alarms may induce desensitization and alert dismissal^29^. Although the function is to hint at potential medical complications, overly sensitive alarms may paradoxically lead to risks to patient safety due to desensitization and alert dismissal in critical situations^62^. Therefore, alarm sensitivity is a factor that might have an effect on healthcare professionals’ acceptance of an AI system and should be taken into consideration when designing AI-based systems in order to enhance acceptance and usage of the systems^63^.

Furthermore, differences in AI acceptance between various occupational groups is a factor that could influence the acceptance of an AI system in a healthcare setting. In this review, we observed a tendency of respondents to perceive AI-based systems more negatively if one’s own professional group was using the AI system rather than another professional group^39^. We could not find more information to back up this theory in the literature. It would therefore be interesting to follow up if the use of the AI system by one’s own professional group does indeed affect his or her perception of the system.

Human factors such as personality and experience were found to affect the perception of an AI system. Depending on the healthcare professional, their needs and the work environment, the acceptance of an AI system might differ^3^. The same AI system might be perceived as helpful by a person and would therefore be accepted while another professional might find that the system could hold up their work and would therefore deem it as unacceptable^3^. Moreover, as found in our review and supported by the literature, more experienced healthcare professionals tend to trust their knowledge and experience more than an AI system. Consequently, they might override the system’s recommendations and make their own decisions based on their personal judgement^3^. This might be related to their fear of losing autonomy in a situation where the AI system is recommending something that is not in line with their critical thinking process.

In addition, time and staff resources are factors that could potentially affect the acceptance of AI systems in healthcare. These factors were perceived differently by different disciplines. With regards to robotic-assisted surgery, medical staff anticipated an increase in operating time and the diagnostic process^39^. Other studies reported that 89.4% of users expected an increase in workload when using a machine-learning algorithm in their clinical routines^32^. Moreover, physicians are often under time constraints during their visits to patients and are overloaded with documentation work. Therefore, they might accept an AI system such as a CDSS if they witness that it might reduce their workload and assist them^3^. In order to facilitate the acceptance and thus implementation of AI systems in clinical settings, it is of utmost importance to integrate these systems into clinical routines and workflows, thereby allowing to reduce the workload.

Interestingly, AI-based systems for the support of the diagnostic process seem to be more established in radiology than among other medical disciplines^30^. This indicates differences in the levels of AI acceptance among healthcare professionals between medical specialties. In implementation studies with reference to AI in radiology, transformative changes with regards to improvements in diagnostic accuracy and value of image analysis were reported^64,65^. This raises the question of whether healthcare professionals in the area of radiology are more technically inclined and specialize on the basis of this enhanced interest or whether innovations of AI in radiology are more easily and better integrated into existing routines and are therefore more widely established and accepted as reported by Recht and Bryan (2017)^64^ and Mayo and Leung (2018)^65^. Furthermore, insufficient knowledge of the limits and potentials of AI technologies’ use may impact healthcare professionals’ acceptance negatively^8,11^. However, as cited many times in the literature, a former introduction to the technology as well as proper training and education on the correct usage of AI might encourage users to accept this technology within their field^3,7,8,13,66,67^. Moreover, transparency in AI data processing is of utmost importance when AI is introduced to healthcare. If the user is able to acknowledge the benefit of the technology and comprehends what AI-based recommendations are based upon, his or her acceptance towards it increases^13,15,68,69^. On the other hand, when the user perceives the use of the AI technology as a threat then his or her level of acceptance decreases^68^. Based on a study reporting the effects of training on acceptance of an AI-based system, it can be stated that the number of training correlated positively with the percentage of participating nurses using the system^47^. In medical education, the necessity to provide training in AI beyond clinical and biomedical skills is emphasized^70,71^. Nonetheless, training requires time and several studies have reported that healthcare professionals lack the time outside their official duty hours to learn how to use new AI-based technologies^7,8,15,68^. Thus, it is an organizational duty to not only offer training for potential users of the AI systems but also to provide staff with timely resources to take part in this training to foster AI acceptance. Furthermore, it should be discussed whether trainings in AI should be integrated early into the educational curriculum^72,73^. Kolachalama and Garg (2018) emphasize the need to integrate expertise from data science and to focus on topics of literacy and practical guidelines in such trainings^71^. Nevertheless, intrinsic motivation to participate in training may also contribute to the seemingly positive effects of the training on the use behaviour observed in the study by Alumran et al. (2020)^47^.

It is important to note that we were not able to replicate the findings of the effect of gender on technology acceptance as proposed by the UTAUT model. In contrast to the UTAUT model, we argue that in this case, there is probably no effect of gender on AI acceptance. However, with regard to age, contradictory results were reported both in our review as well as in the literature. For example, two studies from the literature showed that age impacts trust in AI and that the younger generation leans more toward trusting AI systems than their older counterparts^74,75^. On the contrary, a study by Choudhoury and Asan (2022)^76^ revealed that age did not play a significant role in trusting or intending to use AI.

Nevertheless, training and providing adequate infrastructure with respect to technical support and internet access were unanimously found to be facilitating factors for the acceptance and implementation of AI-based systems in the hospital context and should therefore be considered by the management levels of hospitals^1,13^. To continue, especially with reference to alert systems, aspects such as the alert sensitivity of an AI system and potential consequences in case of elevated sensitivity levels such as alert fatigue and alert dismissal should be kept in mind when determining the safety of a system^61,63^. In order to design a user-friendly AI-based system and enhance its acceptance, it is of utmost importance to involve healthcare professionals early on in the designing stages of the system^77^. We recommend the implementation of user-centred design^78^ during the development of an AI system in healthcare, which would allow the involvement of healthcare professionals in the different stages of the development and evaluation of a system. By incorporating the abilities, characteristics and boundaries of healthcare professionals, the development would result in a secure, uncomplicated and effective AI system. This resulting system would receive high acceptance rates because of healthcare professionals participating in its creation and its integration into clinical routines and workflows would be uncomplicated. Moreover, we also propose longer and intensive research to understand how AI as a complex intervention affects work processes and how people react to it and behave with it. A better understanding of AI-assisted work and decision-making processes could thus be continuously incorporated and the further development of AI systems would profit from it. Finally, in order to facilitate usability and intuitive handling of AI in clinical routine, we recommend to implement training in regards to the theoretical basics, ethical considerations and limitations in view of AI as well as practical skills of usage as early as in undergraduate education.

Reasons for the limited acceptance among healthcare professionals are manifold: Personal fears related to a loss of professional autonomy, lack of integration in clinical workflow and routines, overly sensitive settings for alarm systems, and loss of patient contact are reported. Also, technical reservations such as unintuitive user interfaces and technical limitations such as the unavailability of strong internet connections impede comprehensive usage and acceptance of AI. Hesitation to accept AI in the healthcare setting has to be acknowledged by those in charge of implementing AI technologies in hospital settings. Once the causes of hesitation are known and personal fears and concerns are recognized, appropriate interventions such as training, reliability of AI systems and their ease of use may aid in overcoming the indecisiveness to accept AI in order to allow users to be keen, satisfied and enthusiastic about the technologies.

## Methods

An integrative review of the acceptance of AI among healthcare professionals in the hospital setting was performed. The review protocol was registered in the PROSPERO Database (CRD42021251518). Integrative reviews allow us to reflect on and assess the strength of scientific evidence, identify particular clinical issues, recognize gaps in the current literature, and evaluate the need for further research. An integrative review is based on prior extensive research on a specified topic by means of a literature search^79^. This type of review is of complex nature which makes it prone to the risk of bias. To reduce bias, specific methods are required. Therefore, this review is based on the methodological framework proposed by Whittemore and Knafl^80^. Initially, the topic of interest and the significance of the review is identified. Then, the literature is explored systematically according to a set of identified eligibility criteria. After that, relevant inputs from the included studies are extracted and their quality is appraised. Finally, the outcomes of the studies included in this review are presented and relevance and recommendations for future research are consequently made.

The results of the reviewed articles are presented based on the unified theory of acceptance and use of technology (UTAUT). This theory explains a user’s intention to use information technology systems. It is based on various information technology acceptance models, one of them being the technology acceptance model (TAM)^40^. The UTAUT consists of four main aspects: performance expectancy, effort expectancy, social influences, and facilitating conditions, next to four regulating factors: gender, age, experience and voluntariness of use, which affect the four main aspects^40^ (Table 2).

**Table 2:** Characteristics of the aspects of the Unified Theory of Acceptance of Use of Technology (Venkatesh et al., 2003)^40^.

*The four main aspects of the UTAUT*	
Performance expectancy	characterizes the user’s confidence that using technology will benefit his work performance.
Effort expectancy	represents a user’s beliefs of how easy it is to use the system.
Social influence	describes how much the user feels that significant others believe that they should use the technology.
Facilitating conditions	represents the degree to which the user believes that there exists organizational and technical support to use the system.
*Regulating factors*	
Gender	
Age	
Experience	the user´s familiarity with the system, is thought to affect effort expectancy, social influence and facilitating conditions.
Voluntariness of use	which clarifies whether the system is mandatory or voluntary, is proposed to impact social influence.

### Data collection

Data were sought from records in various databases and grey literature sources. We systematically searched the databases MEDLINE via PubMed, Cochrane Library via Wiley Interscience, Embase and ScienceDirect via Elsevier, Institution of Electrical and Electronics Engineers (IEEE) Xplore via IEEE, Web of Science via Clarivate Analytics, as well as the Cumulative Index to Nursing and Allied Health Literature (CINAHL) via EBSCO for qualitative, quantitative and mixed methods studies. Furthermore, grey literature was searched by means of the dissertation databases Bielefeld Academic Search Engine via BASE, ProQuest, Technische Informationsbibliothek (TIB) as well as the DART Europe E-Theses Portal.

Studies that align with the aim of this study and its research questions were searched for. Keywords were joined using Boolean terms, medical subject headings, and truncation. In close collaboration with a librarian from the local medical university library, the following search string was generated: (Artificial Intelligence OR Machine Learning OR Deep Learning OR Neural Network OR Technol* System OR Smart System OR Intelligent System OR Assistive System OR Decision Support System OR Human–Computer Interaction OR Human Machine Interaction OR Cognitive System OR Decision Engineering OR Natural Language Understanding) AND (Approval OR Intention to Use OR Acceptance OR Adoption OR Acceptability) AND (Nurse OR Doctors OR Physician OR MD OR Clinician OR Healthcare professional OR Healthcare OR Healthcare Worker) AND (Hospital OR Acute Care OR Inpatient care OR Standard Care OR Intensive Care OR Intermediate Care OR Ward). In a subsequent phase, google scholar forward citation tracking was applied to articles included in the database search.

### Inclusion criteria

Quantitative, qualitative and mixed methods original studies published from 2010 up to and including June 2022, in which participants are healthcare professionals and whose clinical fields of work are directly affected by AI (e.g., physicians, nurses, pharmacists, imaging technicians, physiotherapists) were assessed and explored. Studies written in English or German and investigating factors of AI acceptance were considered for review. Other inclusion criteria included studies taking place in hospital settings and studies that describe the development of AI systems with the involvement of healthcare professionals.

### Exclusion criteria

Studies, in which participants were care recipients and family members as well as studies taking place in ambulatory settings, hospices, nursing homes or rehabilitation centres were excluded.

### Screening and extraction process

All studies that resulted from the search were exported to the RAYYAN software, which was used for the screening process^48^. Duplicates were deleted. The remaining research articles were screened separately by two independent reviewers based on title and abstract (M.M. and S.L.). Conflicts between the reviewers were resolved through discussion. The eligibility of relevant studies was appraised based on independent full-text reading by the same two authors. If assessed differently, conflicts were discussed. An extraction table was created by the two reviewers to gather and extract data from the included studies (Table 3).

**Table 3:** Characteristics of included studies and quality appraisal scores.

Author(s) and year	Nature/form of AI	Participant age	Participant’s profession	Sample size	Study design and method	MMAT score	Barriers	Facilitators
Blanco et al. (2018)^26^	CDSS	Not applicable (n.a.)	Nurses, physicians, pharmacists, radiology technicians and environmental services workers	34 (interviews); 13 (survey)	Qualitative semi-structured interviews and surveys	5	Sensitive systems induce alert fatigue	
Catho et al. (2020)^37^	CDSS	n.a.	Physicians	29	Qualitative semi-structured interviews	5	Reduction in time spent with patients	
Chow et al. (2015)^44^	CDSS	n.a.	Physicians	11 (focus group discussions); 265 (survey)	Mixed-methods focus groups and survey	4	Junior physicians were more likely to follow the systems recommendation than senior physicians	
Tscholl et al. (2018)^43^	Monitoring System	35–44 years old	Physicians	128 (interviews); 38 (online survey)	Mixed-methods Interviews and survey	5	Lack of precision in the representation of the information	Visibility of information at a glance enables to interpret the patients‘ situation more quickly
Liberati et al. (2017)^25^	CDSS	n.a.	Physicians, nurses, managers, IT staff	30	Qualitative semi-structured interviews and surveys	5	Lack of understanding of functionalities	
Elahi et al. (2020)^46^	Prognostic model	n.a.	Physicians	25 (questionnaires); 11 (interviews)	Mixed-methods survey and semi-structured interviews	5	Infeasibility of the system if dependent on a strong internet connection	objective assessment of patient risk and support difficult triage decisions, particularly in resource-limited settings
English et al. (2017)^28^	CDSS	25–61 years old	Pharmacists	25	Quantitative survey	4		Facilitating conditions influence clinical pharmacists’ use of the system
Fan et al. (2020)^15^	Medical diagnosis support system	Average age 40 years old	Healthcare professionals in the medical imaging department	191	Quantitative survey	4		
Grau et al. (2019)^27^	CDSS	n.a.	Physicians	21	Qualitative semi-structured interviews	5	Sensitive systems induce alert fatigue	
Hand et al. 2018^82^	CDSS	n.a.	Physicians, nurses and allied health professionals	39	Quantitative survey	4		17/37 (45.9%) felt it would help improve clinician satisfaction 31/35 (88.6%) indicated that they were willing to always or often use the CDSS for fertility discussions
Hsiao et al. (2013)^83^	Pain management decision support systems	n.a.	Nurses	101	Quantitative survey	3		perceived ease of use and perceived usefulness account for 64% of the total explained variance in nurse anaesthetists’ acceptance of PM-DSS.
Jauk et al. (2021)^32^	CDSS	26–42 years old	Physicians and nurses	47 (questionnaires); 15 (expert group)	Mixed-methods interviews & survey	4	14.9% of participants did not believe that the application can be used to detect delirium at an early stage	
Kanagasundaram et al. (2016)^29^	CDSS	n.a.	Physicians	24	Qualitative interviews	5	Alert fatigue System was cited to be an insult to knowledge Workflow interruption	
Khong et al. (2015)^34^	CDSS	Average age junior nurses: 29,8 years old and average age senior nurses 45,5 years old	Nurses	14	Qualitative semi-structured interviews	5	Worry that too much trust in the system might lead to over-reliance and limit the development of clinical skills Participants doubted systems’ accuracy	
Kitzmiller et al. (2019)^41^	Predictive analytics	n.a.	Physicians and nurses	22	Qualitative semi-structured interviews	5	Distal and inconvenient location was perceived to negatively affect routine engagement with the system	
Horsfall et al. (2021)^22^	AI in surgery	31–61 years old or older	Physicians and nurses	100 for quantitative survey, 33 for qualitative	Mixed-methods survey	5		85% of participants strongly or somewhat agreed to the use of AI to enhance real-time alert of hazards or complications
Liang et al. (2019)^35^	Robots	30–36 years old	Nurses	23	Qualitative Semi-structured interviews	3	Fear of a loss of job	Perceived to be ideal for performing repetitive actions, routine tasks and assisting with precision treatment Robotics could also be a useful tool in multi-language communication with children and family caregivers from foreign countries, improving their understanding of the healthcare situation
Lin et al. (2021)^84^	AI in precision medicine	21–40 years old	Physicians and nurses	245 nurses and 40 physicians	Quantitative survey	4		The most dominant determinant for acceptance was perceived usefulness of the system
McBride et al. (2019)^39^	Robots	18 to over 50 years old	Physicians, nurses and support staff	164	Quantitative survey	4	Most participants had concerns about care and handling (*p* = 0.056) Nursing (52.6%) and medical staff (59.6%) were concerned that robotic-assisted surgery will add significant cost and financial pressure on the facility	Most of the nursing, medical and support staff agreed that theoretical, practical training, educational guides and staff support would facilitate the introduction of new technology in the workplace
Norton et al. (2015)^52^	CDSS	<39 to more than 50 years old	Physicians and nurses	32	Quantitative Survey	4	Nonsurgeons reported that the tool would make their job easier more so than surgeons	
								Good educational training tool for residents
Oh et al. (2016)^23^	CDSS	n.a.	Physicians and pharmacists	98	Mixed-methods survey	4	Self-reported lower likelihood to change certain behaviours	
O’Leary et al. (2014)^31^	Clinical pathway support system	n.a.	Physicians, nurses and physiotherapists	19	Mixed-methods Interviews and Surveys	4		Over half of the participants felt that clinical pathway support systems could help the reductions of errors
Omar et al. (2017)^38^	CDSS	n.a.	Physicians	n.a.	Qualitative Semi-structured interviews	1	Some junior nurses preferred to seek advice from senior nurses rather than AI	
Esmaeilzadeh et al. (2015)^85^	CDSS	n.a.	Physicians	335	Quantitative survey	4	Significant relationship between perceived threat to professional autonomy and intention to use CDSS (*β* = −0.392, *p*-value = 0.000)	
Petitgand et al. (2020)^21^	CDSS	n.a.	Physicians	20	Qualitative semi-structured Interviews	5	Systems may favour errors	
Sandhu et al. (2020)^45^	Machine learning	n.a.	Physicians and nurses	15	Qualitative Semi-structured Interviews	5	Unfamiliarity with the system resulted in confusion and misunderstanding	Most useful for residents still developing clinical skills or low-resource community settings
Schulte et al. (2020)^50^	Automatic speech recognition	Mean age of 41.8 ± 9.8 years	Physicians	185	Quantitative Survey	4		Voice recognizer without headset
Stifter et al. (2018)^51^	CDSS	21–71 years old	Nurses	60	Quantitative Survey	4	Higher acceptability among participants with less than one year of experience than those with 1 or more years of experience	
Walter et al. (2020)^53^	Automated pain recognition	Mean age of 40.31 years ± 11.5	Physicians and nurses	102	Quantitative Survey	5		Pain detection accuracy of > 80%
Yurdaisik and Aksoy (2021)^30^	AI	n.a.	Physicians, technicians and medical students	204	Quantitative Survey	4	Only 5.3% of participants stated that they will assume the legal responsibility of imaging results	Among the participants, 51.9% think that AI applications will save time for radiologists
Zheng et al. (2021)^33^	AI in ophtalmology	Less than 25 to older than 45 years old	Physicians and technicians	562	Quantitative Survey	4	56.4% said that in the current ophthalmic AI practice, medical responsibilities are unclear	
Aljarboa et al. (2019)^18^	CDSS	25–51 years old	Physicians	12	Qualitative Semi-structured interviews	5		Alerts direct attention to important issues
Jones et al. (2022)^54^	CDSS	29–62 years old	Physicians and nurses	33	Qualitative Interviews	5	Sensitive systems induce alert fatigue	
Panicker and Sabu (2020)^36^	Computer-assisted medical diagnosis system	27–58 years old	Physicians and technicians	18	Qualitative Interviews	5	Participants doubted systems’ accuracy	
So et al. (2021)^42^	AI	25 years old to 55 or older	Physicians, nurses, pharmacists, physiotherapists and technicians	96	Quantitative Survey	5		Working experience significantly favoured use of AI
Strohm et al. (2020)^86^	AI in radiology	n.a.	Physicians	25	Qualitative Semi-structured interviews	5	Unresolved question of legal responsibility for damage occurred due to e.g. false negatives and false positives resulting from an AI-generated diagnosis	
Pumplun et al. (2021)^49^	Machine learning	n.a.	Physicians, professionals in administrative roles	22	Qualitative Interviews	5	Lack of transparency Limited resources Uncertainties in governmental regulations, strict requirements for the protection of sensitive patient data, and existing medical ethics	
Prakash and Das (2021)^19^	CDSS	82% younger than 40 years old	Physicians	n.a.	Mixed-methods interviews and surveys	5	Lack of understanding of functionalities	
Zhai et al. (2021)^87^	AI	18 to more than 50 years old	Physicians and medical students	307	Mixed-methods Survey	5		
Aljarboa and Miah (2021)^24^	CDSS	25–51 years old	Physicians	54	Qualitative interviews	5		Importance of privacy and security factors as confidentiality and privacy of patient data is essential for use
Nydert et al. (2017)^20^	CDSS	n.a.	Physicians	17	Qualitative interviews	5	Risk of overreliance on the system; double-check of recommended dosage is needed	Greatest benefit within emergency care
Alumran et al. (2020)^47^	Electronic triage and acuity scale	n.a.	Nurses	71	Quantitative survey	5		The years of nurse’s experience influenced their usage of the E-CTAS. There was a positive correlation between years of experience likelihood to become an E-CTAS user

### Quality appraisal

The quality of all included articles were critically assessed by means of the Mixed Methods Appraisal Tool (MMAT) by two authors (M.M. and S.L.)^81^. The MMAT assesses the study quality on the basis of five quality criteria. These criteria include the appropriateness of the research question, of the data collection methods and of the measurement instruments. Ultimately, each study attains a score from zero to five. The higher the score attained, the greater the quality of the appraised study^81^.

Quality appraisal of studies included in integrative reviews improves rigour and diminishes the risk for bias^80^.

### Future directions

Most studies assessed the age of participants. Unfortunately, just four studies assessed the correlation between participant age and levels of acceptance whereof only two observed an effect of age on AI acceptance. In view of the UTAUT model which assumes an effect of age on technology acceptance, it would be of interest to see whether the UTAUT still represents current findings in technology acceptance. Since its publication, the development and use of technology in the wider population have increased substantially. It cannot be ruled out that the availability and integration of technology in the broader population may alter the influence of factors such as age defined in the UTAUT. As a consequence, it would be of interest to re-evaluate the UTAUT model.

### Limitations

We found mixed findings with respect to different AI systems. Most studies addressed CDSSs. It can be argued that by including different types of AI-based systems in the study, interfering variables due to differential proceedings in the handling and function of the systems may have distorted the reported results. It would be of interest to investigate differential hindering and facilitating factors for the acceptance of AI for different kinds of AI-based systems.

In this integrative review, various perspectives of healthcare professionals in hospital settings regarding the acceptance of AI were revealed. Many facilitating factors to the acceptance of AI as well as limiting factors were discussed. Factors related to acceptance or limited acceptance were discussed in association with the characteristics of the UTAUT model. After reviewing 42 studies and discussing them in rapport with studies from the literature, we conclude that hesitation to accept AI in the healthcare setting has to be acknowledged by those in charge of implementing AI technologies in hospital settings. Once the causes of hesitation are known and personal fears and concerns are recognized, appropriate interventions such as training, reliability of AI systems and their ease of use may aid in overcoming the indecisiveness to accept AI in order to allow users to be keen, satisfied and enthusiastic about the technologies.

### Reporting summary

Further information on research design is available in the Nature Research Reporting Summary linked to this article.

## Supplementary information

Reporting Summary

## Data Availability

The data that support the findings of this study are available from the corresponding authors upon reasonable request.
