# Author Correction: An integrative review on the acceptance of artificial intelligence among healthcare professionals in hospitals

**DOI:** 10.1038/s41746-023-00874-z

**Published:** 2023-07-11

**Authors:** Sophie Isabelle Lambert, Murielle Madi, Saša Sopka, Andrea Lenes, Hendrik Stange, Claus-Peter Buszello, Astrid Stephan

**Affiliations:** 1 AIXTRA—Competence Center for Training and Patient Safety, Medical Faculty, RWTH Aachen University, Pauwelsstraße 30, 52074 Aachen, Germany; 2 Department of Anesthesiology, Uniklinik RWTH Aachen, Pauwelsstraße 30, 52074 Aachen, Germany; 3 Department of Nursing Science, Uniklinik RWTH Aachen, Pauwelsstraße 30, 52074 Aachen, Germany; 4 Fraunhofer Society for the Advancement of Applied Research. Fraunhofer-Institute for Intelligent Analysis and Information Systems IAIS, Schloss Birlinghoven 1, 53757 Sankt Augustin Bonn, Germany; 5 Fliedner University of Applied Sciences, Geschwister-Aufricht-Straße, 940489 Düsseldorf, Germany

**Keywords:** Medical ethics, Health policy, Public health

Correction to: *npj Digital Medicine* 10.1038/s41746-023-00852-5, published online 10 June 2023

In the Author contributions section, the sentence SL wrote the manuscript should read as SL and MM wrote the manuscript’. The original article has been corrected.

